# Psychosocial and demographic factors influencing pain scores of patients with knee osteoarthritis

**DOI:** 10.1371/journal.pone.0195075

**Published:** 2018-04-09

**Authors:** Lauren Eberly, Dustin Richter, George Comerci, Justin Ocksrider, Deana Mercer, Gary Mlady, Daniel Wascher, Robert Schenck

**Affiliations:** 1 Department of Orthopaedics & Rehabilitation, The University of New Mexico Health Sciences Center, Albuquerque, New Mexico, United States of America; 2 Department of Internal Medicine, The University of New Mexico Health Sciences Center, Albuquerque, New Mexico, United States of America; 3 Department of Radiology, The University of New Mexico Health Sciences Center, Albuquerque, New Mexico, United States of America; Augusta University, UNITED STATES

## Abstract

**Background:**

Pain levels in patients with osteoarthritis (OA) of the knee are commonly assessed by using a numeric scoring system, but results may be influenced by factors other than the patient’s actual physical discomfort or disease severity, including psychosocial and demographic variables. We examined the possible relation between knee-pain scores and several psychosocial, sociodemographic, disease, and treatment variables in 355 patients with knee OA.

**Methods:**

The pain-evaluation instrument was a 0- to 10-point rating scale. Data obtained retrospectively from the patients’ medical records were demographic characteristics, body mass index (BMI), concomitant disorders, illicit and prescription drug use, alcohol use, smoking, knee OA treatment, and severity of knee OA indicated by Kellgren-Lawrence (KL) radiographic grade. Univariate and multivariate analyses were performed to determine whether these variables correlated with reported pain scores.

**Results:**

On univariate analysis, higher pain scores were significantly associated with Native American or Hispanic ethnicity; a higher BMI; current prescription for an opioid, antidepressant, or gabapentinoid medication; depression; diabetes mellitus; fibromyalgia; illicit drug use; lack of health insurance; smoking; previous knee injection; and recommendation by the clinician that the patient undergo knee surgery. Neither the patient’s sex nor the KL grade showed a correlation. On multivariate analysis, depression, current opioid prescription, and Native American or Hispanic ethnicity retained a significant association with higher pain scores.

**Conclusions and implications:**

Our results in a large, ethnically diverse group of patients with knee OA suggest that psychosocial and sociodemographic factors may be important determinants of pain levels reported by patients with knee OA.

## Introduction

More than nine million people in the United States have symptomatic knee osteoarthritis (OA) [1], with pain being the primary reason patients seek care and the leading cause of disability from the disease [2,3]. Patients who present with OA are usually asked to describe their level of pain, often by referencing a numeric rating scale. However, a discrepancy between patients’ reports of their pain level and OA disease severity as assessed by radiographic studies has often been observed [4–6]. Therefore, it has been suggested that demographic and psychosocial factors may influence pain reports.

Factors that have been assessed for their possible relation to reports of OA-associated pain include age, sex, body mass index (BMI), race/ethnicity, substance abuse, and psychological variables such as depression, hopelessness, overall well-being, and social stress [4,7–10]. The results of such correlation studies have varied, however, and some factors that may affect pain-level reports have not been examined. With the goal of increasing understanding of determinants of pain reports in patients with knee OA, we conducted a retrospective study of the possible correlation between pain score and several demographic, psychosocial, disease, and treatment characteristics, as well as between pain score and the severity of OA as assessed radiologically with use of the Kellgren-Lawrence (KL) classification system [11]. In addition, because little research has been done on a possible link between patients’ reported pain levels and clinical decision making, we examined whether pain scores were correlated with a recommendation by the surgeon that the patient undergo surgical rather than nonoperative treatment of OA. Our hypothesis was that demographic, psychosocial, and overall health-status variables would correlate with patients’ reported levels of pain.

## Materials and methods

### Patients

With approval from the Human Research Review Committee of The University of New Mexico Health Sciences Center (HRRC#13–523), we reviewed the medical records of all 611 new patients who presented to our orthopaedics clinic and received a primary diagnosis of knee OA between 1st January 2013 and 31st December 2013. In each case, the diagnosis was confirmed by an evaluation of the patient’s medical history, a physical examination, radiographic studies, or a combination of these methods. Patients with a concurrent ligamentous injury, inflammatory arthritis, or bilateral knee OA were excluded from the study; therefore, 355 were enrolled. Institutional review board waived the need for patient consent, and data was accessed anonymously with no patient identifiers.

At their initial presentation, all patients had been asked to describe their pain level with respect to a number from 0 to 10, with 0 representing “no pain” and 10 “the worst pain imaginable.” This numeric scale is commonly used to assess arthritic pain [12]. Aside from pain score, the record review obtained the following data for each patient: age; sex; race or ethnicity (self-reported by patients’ checking a box on intake form); BMI; current tobacco, alcohol, and illicit drug use (both illegal drugs and overuse of legal drugs); current prescription for an opioid, gabapentinoid, or antidepressant agent; current diagnosis of depression, fibromyalgia, or posttraumatic stress disorder (PTSD); health-insurance status (yes or no); previous injection of a corticosteroid agent or hyaluronic acid in the affected knee; and whether the patient’s clinician recommended surgical treatment of the knee OA during the initial presentation. Information on smoking, alcohol, and illicit drug use was obtained with a self-report intake form that did not allow specification of the level of use or the type of illicit drug. BMI was calculated from patients’ most recently documented height and weight measured at that clinic visit (S1 Dataset).

At the time of the medical-record review, radiographs obtained during the initial patient visit were assessed by a musculoskeletal radiologist who was blinded to all patient information in the records, including pain scores. The radiologist assigned each image a KL grade of 0, 1, 2, 3, or 4 on the basis of the extent of degenerative changes observed.

### Statistical analysis

In estimating the appropriate sample size for the study, we assumed that most clinicians would consider a difference in pain score of 2 to be clinically important and we wanted to limit the type I error to 0.05 or lower and achieve a power of 90%. Because we anticipated simultaneous analysis of up to 22 independent variables, we used a type I error of 0.002 (on the basis of the Bonferroni inequality). Under these assumptions, we calculated that the study should include about 310 patients.

Standard summary statistics were calculated for all variables. Univariate analysis was used initially to assess the possible relation between pain score and each independent binary variable studied. The Student t test or Mann-Whitney U test was applied as appropriate. Analysis of variance was used to examine the possible relation between pain score and each of the following: age, BMI, race or ethnic group, and KL grade. A p value of <0.05 was considered to indicate a statistically significant difference.

Multivariate analysis was performed by using a general linear model algorithm and maximum-likelihood estimation. Variables that were significantly associated with pain score in the univariate analysis were sequentially fitted into the model with use of adjusted R^2^ analysis. The variables were removed sequentially until only those that had a significant relation with pain score remained in the model. All statistical analyses were performed with Statgraphics Centurion XV software (StatPoint, Herndon, VA).

## Results

### Patient characteristics

Table 1 shows characteristics of the 355 patients in the study. Most of the patients were white women, but our cohort was also diverse in that Native American and Hispanic patients each comprised more than 10% of the total. The patients ranged in age from twenty-four to ninety years. The average BMI was 31.0 kg/m^2^ (range, 19.1–61.9 kg/m^2^). The overall mean (standard deviation [SD]) pain score was 5.0 (2.9). The mean pain scores in white, Native American, Hispanic, “other,” and black patients were 4.5 (3.0), 6.3 (2.7), 6.4 (2.5), 4.7 (3.1), and 5.8 (2.8), respectively. The “other” category included two patients who indicated that they were Asian and twenty-seven who reported that they were “other” than white, Native American, Hispanic, or black. The most common KL score was 3, indicating moderate OA.

**Table 1:** Demographic and clinical variables for 355 patients with knee osteoarthritis^a^.

Variable	Value
Age, y, mean ± standard deviation	58.6 ± 11.8
Male	139 (39)
Female	216 (61)
Body mass index, kg/m^2^, mean ± standard deviation	31.2 ± 7.3
Ethnicity^b^	
White	191 (64.7)
Native American	35 (11.9)
Hispanic	30 (10.2)
Other	29 (9.8)
Black	10 (3.4)
Comorbidities/insurance/history	
Current smoking	56 (15.9)
Alcohol use	157 (46.6)
Illicit drug use	18 (5.4)
Opioid-agent prescription	75 (21.6)
Gabapentinoid prescription	43 (12.4)
Antidepressant prescription	84 (24.2)
Depression	94 (27.1)
Fibromyalgia	22 (6.4)
Posttraumatic stress disorder	7 (2.0)
Health insurance	318 (91.4)
Previous knee injection	110 (32.3)
Surgery recommended by clinician	59 (16.8)
Kellgren-Lawrence grade^c^	
0	9 (2.8)
1	46 (14.2)
2	82 (25.4)
3	110 (34.1)
4	76 (23.5)

Values represent number of patients (percentage of the indicated group), unless otherwise stated.

^a^There were no significant differences between the groups.

^b^Race or ethnic group was self-reported.

^c^A Kellgren-Lawrence grade of 0 (no osteophytes or joint-space narrowing) indicates no osteoarthritis; a grade of 1 (questionable osteophytes), possible osteoarthritis; a grade of 2 (definite osteophytes, no joint-space narrowing), mild osteoarthritis; a grade of 3 (≤ 50% joint-space narrowing), moderate osteoarthritis; and a grade of 4 (≥ 50% join-space narrowing), severe osteoarthritis.

### Univariate and multivariate analysis

**Fig 1:**
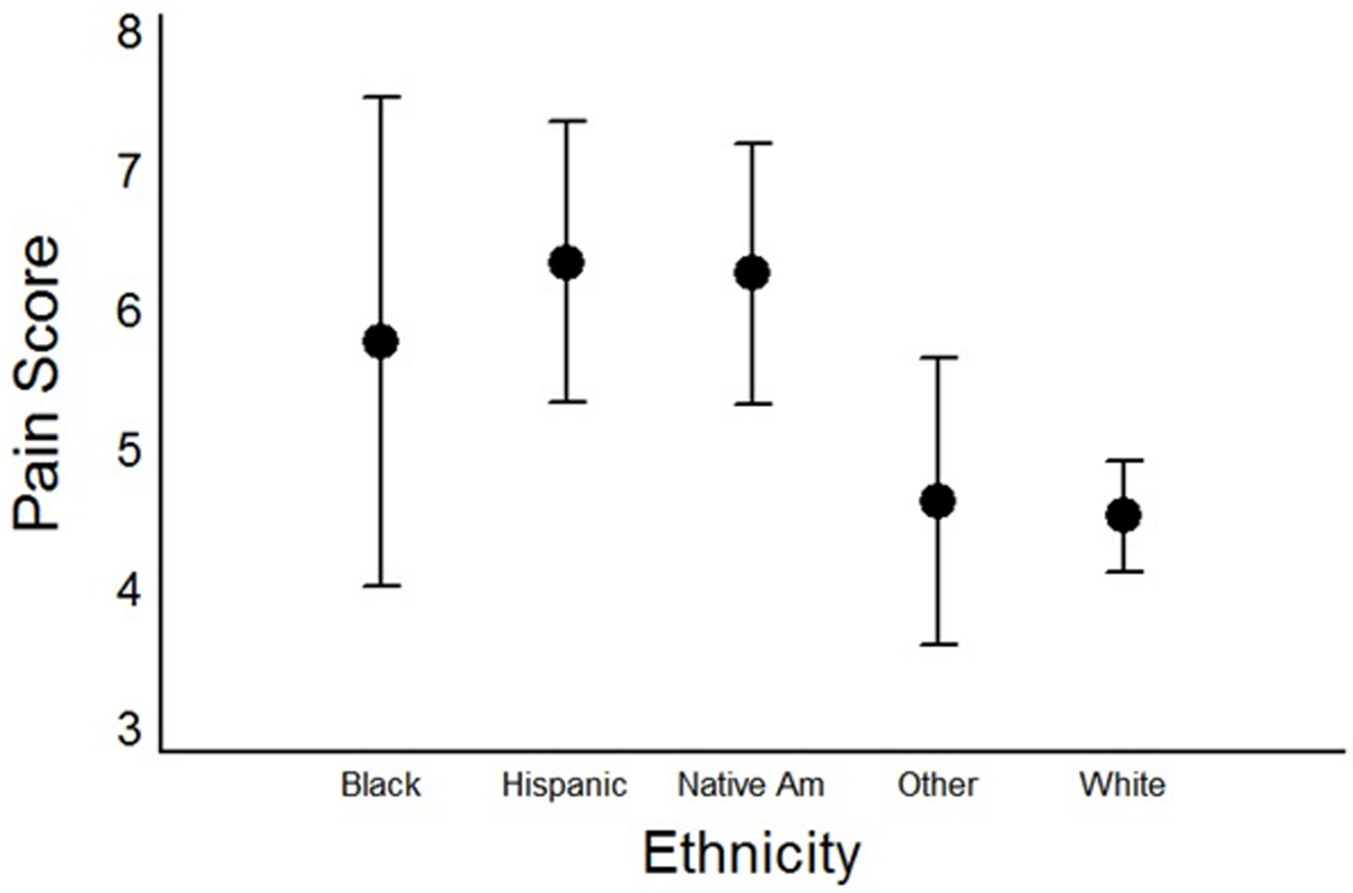
Ethnicity and pain scores. Mean knee-pain scores in 355 patients with knee osteoarthritis, according to patients’ ethnic or racial group. The error bars represent 95% confidence intervals adjusted by using the Tukey procedure for multiple comparisons. The difference between Hispanic and white patients and between Native American and white patients was significant (p < 0.001 for both comparisons).

On univariate analysis, age had a significant inverse relation to pain score, with younger subjects having significantly higher scores (p = 0.03). Compared with white patients, Hispanic or Native American patients had significantly higher pain scores (p < 0.001 for both comparisons; (Fig 1), but there were no other significant differences among racial or ethnic groups. Patients with higher BMIs also had higher pain scores (p < 0.001). With respect to binary variables (Table 2), patients with a current opioid prescription, depression, fibromyalgia, illicit drug use, current antidepressant or gabapentinoid prescription, uninsured status, smoking, previous knee injection, or recommendation for operative treatment of OA had significantly higher pain scores than those without these characteristics. Patients who said that they drank alcohol had significantly lower pain scores than those who said that they did not.

**Table 2:** Association of binary variables and pain score on univariate analysis.

Variable	Mean ± standard deviation score (95% CI)	*P* Value
Sex		
Female	5.00 ± 2.91 (4.61–5.39)	
Male	5.05 ± 2.98 (4.55–5.55)	0.86
Opioid-agent prescription		
Yes	6.84 ± 2.94 (6.36–7.32)	
No	4.48 ± 2.08 (4.13–4.83)	<0.001
Depression		
Yes	6.19 ± 2.52 (5.67–6.71)	
No	4.58 ± 2.94 (4.22–4.95)	<0.001
Post-traumatic Stress Disorder		
Yes	6.14 ± 2.48 (3.85–8.44)	
No	4.96 ± 2.93 (4.64–5.27)	0.29
Fibromyalgia		
Yes	6.77 ± 2.07 (5.86–7.69)	
No	4.86 ± 2.93 (4.54–5.18)	0.003
Illicit drug use		
Yes	6.89 ± 2.58 (5.60–8.18)	
No	4.88 ± 2.91 (4.55–5.20)	0.004
Antidepressant prescription		
Yes	5.76 ± 2.77 (5.15–6.36)	
No	4.73 ± 2.96 (4.37–5.09)	0.005
Gabapentinoid prescription		
Yes	6.14 ± 2.53 (5.36–6.92)	
No	4.82 ± 2.96 (4.48–5.15)	0.006
Alcohol use		
Yes	4.53 ± 2.73 (4.10–4.96)	
No	5.36 ± 3.04 (4.91–5.81)	0.009
Health insurance		
Yes	4.90 ± 2.93 (4.57–5.22)	
No	6.27 ± 2.66 (5.27–7.26)	0.01
Smoking		
Yes	5.82 ± 2.39 (5.18–6.46)	
No	4.85 ± 2.99 (4.50–5.19)	0.02
Previous knee injection		
Yes	5.47 ± 2.94 (4.92–6.02)	
No	4.75 ± 2.92 (4.37–5.13)	0.03
Surgery recommended by clinician		
Yes	5.69 ± 2.94 (4.92–6.02)	
No	4.87 ± 2.92 (4.53–5.20)	0.04

Abbreviation: CI, confidence interval.

**Fig 2:**
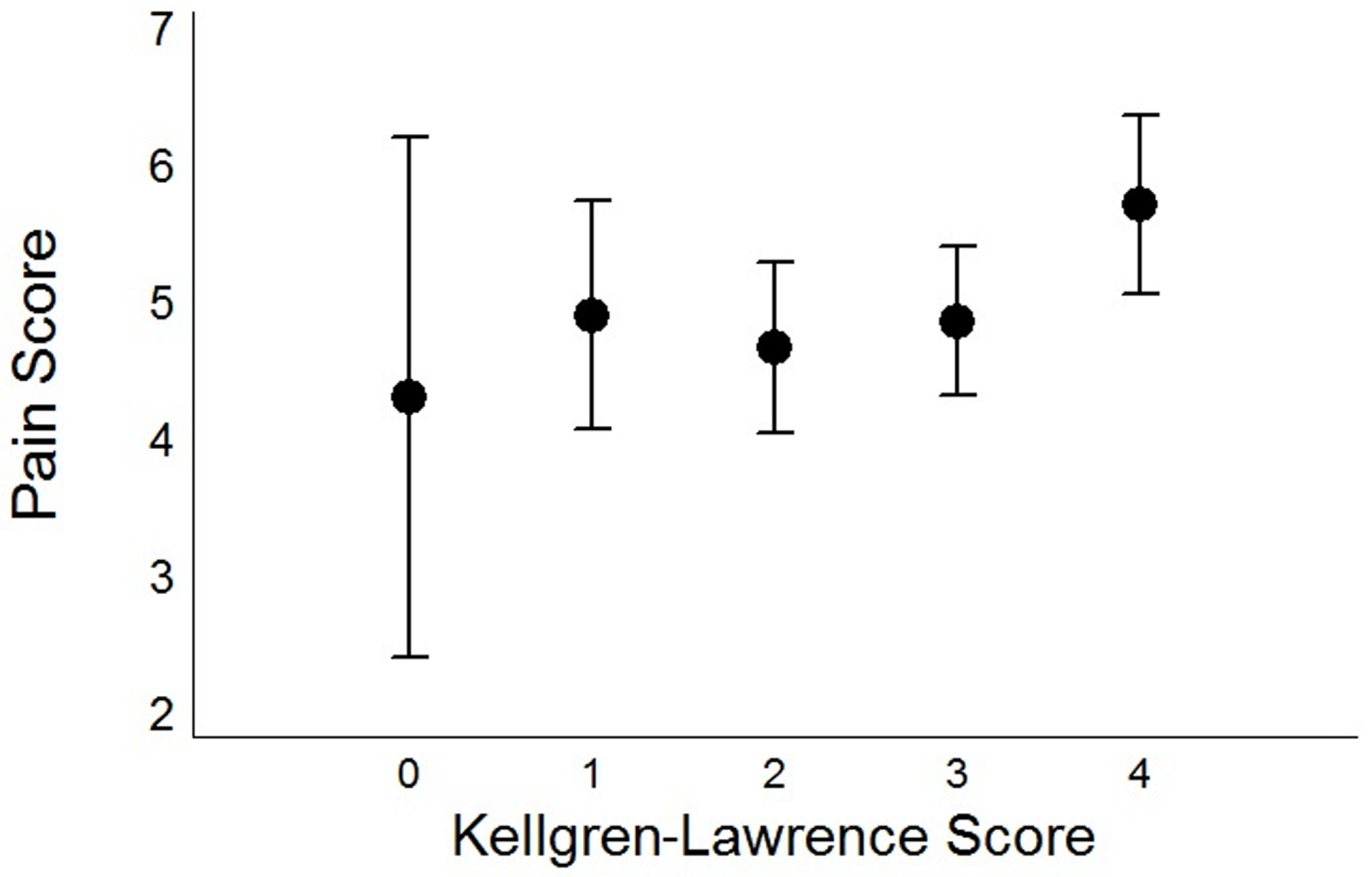
Kellgren-Lawrence grade and pain scores. Mean knee-pain scores in 355 patients with knee osteoarthritis, according to Kellgren-Lawrence Grade. The error bars represent 95% confidence intervals adjusted by using the Tukey procedure for multiple comparisons. There were no significant differences between groups.

The only variables that were not related to reported pain levels were the sex of the patient, a diagnosis of PTSD, and KL grade (p = 0.2; Fig 2). The mean pain scores (SD) according to KL grade were 4.33 (1.89) for grade 0; 4.93 (0.84) for grade 1; 4.70 (0.63) for grade 2; 4.89 (0.54) for grade 3; and 5.74 (0.65) for grade 4.

On multivariate analysis, current opioid prescription, depression, and Native American or Hispanic ethnicity remained in the model, indicating a significant relation between those variables and pain scores (p < 0.001, p = 0.002, and p < 0.001, respectively).

## Discussion

In a univariate analysis of a large, diverse cohort of patients with knee OA, we found that several psychosocial, sociodemographic, disease, and treatment variables were each significantly associated with higher reported knee-pain scores. A subsequent multivariate analysis showed a significant association between higher pain scores and Hispanic or Native American ethnicity, opioid agent prescription, and depression. There was no relation between pain score and KL grade, a discordance also observed in several earlier studies [4–6]. Many of the factors analyzed have not, to our knowledge, previously been correlated with pain score: Hispanic or Native American ethnicity, age, opioid prescription, fibromyalgia, illicit drug use, antidepressant prescription, alcohol use (inverse relation), gabapentinoid prescription, health-insurance status, smoking, previous knee injection, and recommendation for surgical versus nonoperative treatment of knee OA. It is important to note that although the results yielded statistical significance across multiple factors analyzed in this study, the factors with clinical significance, according to our stated criteria of a difference in pain score of at least two, are opioid prescriptions and illicit drug use.

Other research has examined a possible link between knee OA-associated pain and race/ethnicity. A study by Creamer et al. [4] included only black and white patients and noted that the former reported higher pain scores than the latter. Additionally, one of the few studies to include Hispanic patients with rheumatic disease found significantly poorer global estimates of participant health, compared with those made by physicians [13]. The diversity of our study cohort provided an opportunity to compare pain scores among four different racial/ethnic groups. Both our univariate and multivariate analyses found that Native American or Hispanic patients had significantly higher pain scores than white patients.

Studies of arthritis-associated pain in Native Americans are scarce; however, in an investigation by Kramer et al. [14] in which face-to-face interviews were conducted with fifty-six Native Americans living in an urban area, most interviewees commented on their cultural practice of minimizing pain complaints and reported that American Indians do not readily discuss pain. The Native Americans studied by Kramer et al. lived in an urban area, whereas many of our Native American patients live in rural areas and are of different tribes. There may be cultural variations among tribes regarding the pain experience or the reporting of pain in a setting in which few, if any, of the clinicians are Native American. Nevertheless, these studies do show the importance of clinician awareness of possible ethnic-group differences in reporting OA-pain experience.

The association between current opioid agent prescription and higher pain scores possibly stems from neuromodulation of pain sensitivity resulting from chronic opioid use [15,16]. It is possible that some of our patients with an opioid prescription reported greater pain as a manifestation of drug-seeking behavior whereas other patients may simply have higher baseline pain levels. However, the differences in pharmacodynamics, opioid-receptor interactions, and bioavailability of a particular opioid dose likely play an important role in sufficient pain relief. The use of a pain-assessment instrument that evaluates patients’ functional abilities, such as the Western Ontario and McMaster Universities Osteoarthritis Index [17], might be useful when pain assessment is unclear.

Our multivariate analysis also found a significant relation between higher pain scores and depression. Previous studies found that depression affects reported pain in patients with rheumatoid arthritis [18], and an influence of depression on pain severity in patients with OA has been suggested [6,19]. The higher reported pain scores might reflect pain catastrophizing, which is a tendency to focus on negative pain sensations, thereby exaggerating the pain experience and enhancing feelings of helplessness [20]. It was previously shown that depression can lead to increased pain through pain catastrophizing in patients with musculoskeletal conditions [21], and pain catastrophizing has been linked to higher pain scores in patients with OA [22]. Cognitive behavioral therapy or psychotherapy aimed at reducing catastrophizing in these patients and similar patients with fibromyalgia may produce better outcomes than pharmacologic therapy [23].

In agreement with earlier findings [7,8], the sex of the patient was not correlated with pain scores. Interestingly, although the prevalence of knee OA increases with age, pain scores were significantly higher in our study’s younger patients. Psychological factors such as depression may have influenced pain reports in these patients. Indeed, younger adults with OA have been shown to have higher rates of depression and depressive symptoms than older adults with the disease [24,25]. In addition, life stress and hypochondriasis, which are associated with higher pain scores, are more prevalent in younger people [10].

Obesity, a known risk factor for development of OA, has been linked to an increased severity of OA-related pain, although Somers et al. [26] found that BMI was not correlated with pain scale scores. Our results show that patients with higher BMIs were significantly more likely to have higher knee-pain scores. Clinicians counseling obese patients with OA-related knee pain should consider suggesting weight loss as possible treatment.

The association between higher pain scores and uninsured status is probably related to socioeconomic status because OA pain and symptomatic knee OA have been correlated with lower income levels [7] and the poverty rate in a community [27], respectively. In addition, “feeling helpless” has been found to constitute an important determinant of higher pain scores [8], and patients without health insurance may have an increased tendency to experience this feeling, perhaps accompanied by a feeling of disenfranchisement.

Our study had the usual limitations of a retrospective, observational investigation. In addition, there may have been interactions among the variables assessed that our study design could not identify. Some factors not examined, such as whether the patient was currently involved in a workers’ compensation evaluation, might have influenced pain-score reports. Additionally, our study had a small number of black patients, with a large percentage of Hispanic and Native American patients. This unique ethnic distribution, however, is a reflection of our state’s composite population. Finally, the pain-evaluation method that was used was a simple numeric rating scale. This assessment can be administered quickly, but it may not sufficiently characterize a patient’s pain experience.

Despite these limitations, our results indicate that psychosocial and sociodemographic factors significantly affect patients’ reports of their level of OA-related knee pain and that patients with OA do not constitute a homogenous group for which the same management approaches will suffice. Management should be tailored to the individual and characterized by more extensive patient education regarding the specific factors contributing to their pain, as well as discussion of appropriate expectations for pain-level. The usefulness of a pain assessment based primarily on a numeric pain score is of variable utility. Extensive reliance on such an assessment in clinical decision-making may be inappropriate, especially when invasive procedures are contemplated. With the limitations that come with the visual analog scale, clinicians could consider employing various self-reported and physical measures recommended by the Osteoarthritis Research Society International (OARSI) such as the Dallas Pain Questionnaire or the WOMAC Questionnaire. Ultimately, pain-treatment decisions should not be based primarily on KL grade but the entire patient presentation.

Although clinicians cannot modify some of the factors associated with higher pain scores in our study, they can increase their insight into the possible causes of an observed discrepancy between a patient’s clinical appearance with respect to comfort and his or her pain score and perhaps mitigate the influence of factors that can be changed. Clinicians who are aware of the psychosocial and sociodemographic characteristics that affect their patients’ reports of OA-related knee pain will thereby be able to provide more effective, patient-centered care.

## Supporting information

S1 Dataset Knee osteoarthritis patient raw data. Raw data extracted from the chart review for all patients who met study inclusion criteria.
(XLS)
